# muBLASTP: database-indexed protein sequence search on multicore CPUs

**DOI:** 10.1186/s12859-016-1302-4

**Published:** 2016-11-04

**Authors:** Jing Zhang, Sanchit Misra, Hao Wang, Wu-chun Feng

**Affiliations:** 1 Department of Computer Science, Virginia Tech, 225 Stanger Street, Blacksburg, 24060 VA USA; 2 Parallel Computing Lab, Intel Corporation, Bengaluru, Karnataka, 560102 India

**Keywords:** BLAST, Database index, Local alignment, Multicore

## Abstract

**Background:**

The Basic Local Alignment Search Tool (BLAST) is a fundamental program in the life sciences that searches databases for sequences that are most similar to a query sequence. Currently, the BLAST algorithm utilizes a query-indexed approach. Although many approaches suggest that sequence search with a database index can achieve much higher throughput (e.g., BLAT, SSAHA, and CAFE), they cannot deliver the same level of sensitivity as the query-indexed BLAST, i.e., NCBI BLAST, or they can only support nucleotide sequence search, e.g., MegaBLAST. Due to different challenges and characteristics between query indexing and database indexing, the existing techniques for query-indexed search cannot be used into database indexed search.

**Results:**

muBLASTP, a novel database-indexed BLAST for protein sequence search, delivers identical hits returned to NCBI BLAST. On Intel Haswell multicore CPUs, for a single query, the single-threaded muBLASTP achieves up to a 4.41-fold speedup for alignment stages, and up to a 1.75-fold end-to-end speedup over single-threaded NCBI BLAST. For a batch of queries, the multithreaded muBLASTP achieves up to a 5.7-fold speedups for alignment stages, and up to a 4.56-fold end-to-end speedup over multithreaded NCBI BLAST.

**Conclusions:**

With a newly designed index structure for protein database and associated optimizations in BLASTP algorithm, we re-factored BLASTP algorithm for modern multicore processors that achieves much higher throughput with acceptable memory footprint for the database index.

**Electronic supplementary material:**

The online version of this article (doi:10.1186/s12859-016-1302-4) contains supplementary material, which is available to authorized users.

## Background

The Basic Local Alignment Search Tool (BLAST) [1] is a fundamental algorithm in life sciences that compares a query sequence to a database of sequences, i.e., subject sequences, to identify sequences that are the most similar to the query sequence. The similarities identified by BLAST can be used to infer functional and structural relationships between the corresponding biological entities, for example.

With the advent of next-generation sequencing (NGS), whether at the outset or downstream from NGS, the exponential growth of sequence databases is arguably outstripping our ability to analyze the data. Specifically, the increasing demands to mine sequence databases for useful information requires substantial computing power. Consequently, significant research effort has been invested into accelerating the BLAST search algorithm.

Much of this research effort has focused on the parallelization of BLAST on different parallel architectures due to its compute- and data-intensive nature. NCBI BLAST+ [2] uses pthreads to speed up BLAST on a multicore CPU. On CPU clusters, TurboBLAST [3], ScalaBLAST [4], and mpiBLAST [5] have been proposed. To achieve higher throughput on a per-node basis, BLAST has also been mapped and optimized onto various accelerators, including FPGAs [6, 7] and GPUs [8–13]. However, there are few recent studies that focus on improving the performance of CPU implementations of the widely-used BLAST algorithm.

Most previous studies [1, 4, 14, 15] adopt query indexing for sequence search. Query indexing uses a lookup table to record positions of each word in the input query. These BLAST algorithms then scan each database sequence to find short matches, extend these matches to optimal alignments, and then calculate the final similarity scores. In contrast, other approaches suggest that database indexing can yield much faster speed than query indexing [16, 17]. Examples of such tools include BLAT [1], SSAHA [18], MegaBLAST [19], and CAFE [20]. However, these tools cannot provide the same level of sensitivity as the BLAST algorithm [17, 21, 22], or support nucleotide sequence search.

SSAHA and BLAT, for example, are significantly fast for finding near-identical matches. However, to reduce memory footprint and search space, both tools build indexes of non-overlapping words from the database, which leads to extremely fast search but compromised sensitivity. More specifically, BLAT, for example, builds database index with non-overlapping words of length *W*. With this approach, the size of database index is significantly reduced, roughly $\frac {1}{W}$ the size of an index with overlapping words. However, it requires a matching region of 2*W*−1 letters between two sequences for guaranteeing to detect it. CAFE is another search tool supporting protein sequence with database index, but the search method and scoring phase are substantially changed. MegaBLAST is the only BLAST variant based on database index. MegaBLAST accelerates the search for highly similar sequences by using a large word size (W = 28) to reduce the search workload and the memory usage. However, according to the previous studies [23–25], increasing word size could sacrifice the sensitivity and accuracy. Furthermore, MegaBLAST only supports nucleotide sequences, as the authors claimed that it is very challenging to support protein sequence based on their design.

Because query indexing usually contains a high percentage of empty slots due to few letters in a query, most of the optimizations of query indexing seek to reduce the sparsity of the index, e.g., the thick backbone and the position array in NCBI BLAST [26] and the deterministic finite automaton (DFA) in FSA-BLAST [14]. For database indexing, which is full of positions from millions of subject sequences from a database (e.g., about 6 million sequences in *env_nr* database, and over 85 million sequences in *nr* database), the major challenges differ substantially from query indexing. First, the size of the database index can be prohibitive, especially for the protein database, which has the increased alphabet and the short word length. Second, unlike nucleotide sequence search, protein sequence search needs to search the hits of similar words, i.e. the neighboring words rather than merely and exactly matched words. Including neighboring words increases the size of the index by one or two orders of magnitude. Third, BLAST employs input-sensitive heuristics to quickly eliminate unnecessary search spaces. However, this heuristic introduces significant irregularities in memory access patterns and in control flow paths, e.g. during two-hit ungapped extension in protein sequence search. Thus, database indexing that aligns a query to millions of database sequences instead of a single database sequence iteratively will suffer more from such irregularities, leading to serious performance degradation.

To overcome these challenges of database indexing for protein sequence search, we propose muBLASTP (i.e., microprocessor-based BLASTP), a novel BLASTP algorithm that includes an advanced index data structure for sequences of the database and a set of optimizations for the BLASTP algorithm. The experimental results show that on a modern multicore architecture, namely Intel Haswell, for a single query, the single-threaded muBLASTP can deliver up to a 4.41-fold speedup for alignment stages, and up to a 1.75-fold end-to-end speedup over the single-threaded NCBI BLAST. For a batch of queries, the multithreaded muBLASTP can achieve up to a 5.7-fold speedup for alignment stages, and 4.56-fold end-to-end speedup over the multithreaded NCBI BLAST using 24 threads. The experimental results also shows that on a older generation multicore architecture, namely Intel Nehalem, for a single query, muBLASTP still can deliver up to a 3.8-fold speedup for alignment stages, and up to a 1.94-fold end-to-end speedup over the single-threaded NCBI BLAST. For a batch of queries, the multithreaded muBLASTP can achieve up to a 8.59-fold speedup for alignment stages, and 3.85-fold end-to-end speedups over the multithreaded NCBI BLAST using 12 threads. In addition to improving performance significantly, muBLASTP produces identical hit returned to NCBI BLAST, which is important to the bioinformatics community.

## Implementation

### Database index

The most challenging component of muBLASTP is the design of the database index. The index should include the positions of overlapping words from all subject sequences of the database. Thus, each position contains the information for the sequence id and the offset in the subject sequence, i.e., subject offset. For the protein sequence search, the BLASTP algorithm uses the small word size (*W*=3), large alphabet size (22 letters), and neighboring word comparisons. Because these factors may make the database index very large, we design our database index with the following techniques: index blocking, sorting, and compression.

#### Index blocking

**Fig. 1:**
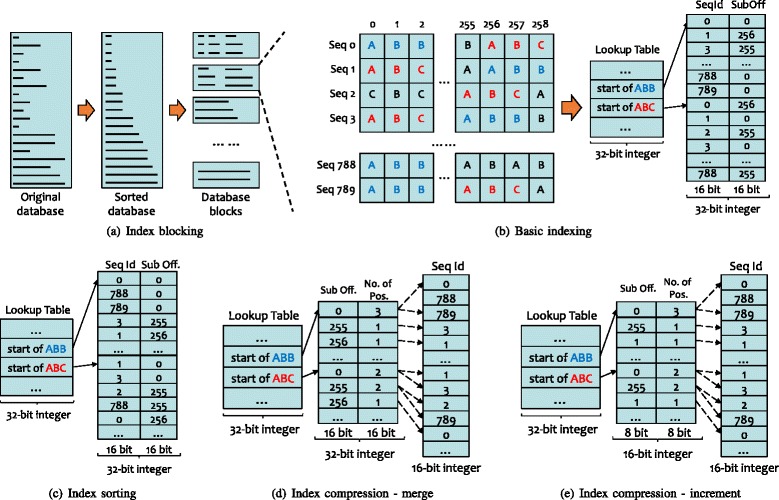
An example of building a compressed database index. The figure shows the flow from the original database to the compressed index. **a** Index blocking phase partitions the sorted database into blocks. **b** Basic indexing phase generates basic index, which contains positions of all words in the database. **c** Index sorting sorts positions of each word by subject offsets. **d** Index compression-merge merges positions with the same subject offset. **e** Index compression-increment done on the merged positions generates increments of subject offsets and sequence ids

Figure 1 a illustrates the design of index blocking. We first sort the database by the sequence length; partition the database into small blocks, where each block has the same number of letters; and then build the index for each block separately. In this way, the search algorithm can go through the index blocks one by one and merge the high-scoring results of each block in the final stage. Index blocking can enable the database index to fit into main memory, especially for large databases whose total index size exceeds the size of main memory. By configuring the size of the index block, we can achieve better performance. For example, if the index block is small enough to fit into the CPU cache, the hit detection and gapped and ungapped extension may achieve better data locality.

Another benefit of using index blocking is to reduce the index size. Without index blocking and assuming a total of *M* sequences in the database, we need log2*M* bits to store sequence ids. After dividing the database into *N* blocks, each block contains $\frac {M}{N}$ sequences on average. Thus, we only need $\log _{2} \lceil \frac {M}{N}\rceil $ bits to store sequence ids. For example, if there are 2^20^ sequences in a database, we need 20 bits to represent the sequence ids. With 2^8^ blocks, if each block contains 2^12^ sequences, then we only need a maximum of 12 bits to store the sequence ids. In addition, because the number of bits for storing subject offsets is determined by the longest sequences in each block, after sorting the database by the sequence length, we can use fewer bits for subject offsets in the blocks having short and medium sequences, and more bits only for the blocks having extremely long sequences. (This is the reason why we sort the database by the sequence length).

Furthermore, index blocking allows us to parallelize the BLASTP algorithm via the mapping of one block to a thread on a modern multicore processor. For this block-wise parallel method to achieve the ideal load balance, we partition index blocks equally to make each block have a similar number of letters, instead of an identical number of sequences. To avoid cutting a sequence in the middle, if this sequence reaches the cap of the block size, we put it into the next block.

After the database is partitioned into blocks, each block is indexed individually. As shown in Fig. 1 b, the index consists of two parts: the lookup table and the position array. The lookup table contains *a* ^*w*^ entries, where *a* is the alphabet size of amino acids and *w* is the length of the words. Each entry contains an offset to the starting position of the corresponding word. In the position array, a position of the word consists of the sequence id and the subject offset. For protein sequence search, the BLASTP algorithm not only searches the hits of exactly matched words, but it also searches the neighboring words, which are similar words. The query index used in existing BLAST tools, e.g., NCBI BLAST, includes the positions of neighboring words in the lookup table. However, for the database index in muBLASTP, if we store the positions for the neighboring words, the total size of the index becomes extraordinarily large. To address this problem, instead of storing positions of the neighboring words in the index, we put the offsets, which point to the neighboring words of every word, into the lookup table. The hit detection stage then goes through the positions of neighbors via the offsets after visiting the current word. In this way, we use additional stride memory accesses to reduce the total memory footprint for the index.

#### Index compression

As shown in Fig. 1 b, a specific subject offset for a word may be repeated in multiple sequences. For example, the word “ABC” appears in position 0 of sequence 1 and 3. In light of this repetition, it is possible to compress the index by optimizing the storage of subject offsets. Next, we sort the position array by the subject offset to group the same subject offsets together, as shown in Fig. 1 c. After that, we reduce the index size via merging the repeated subject offsets: for each word, we store the subject offset and the number of positions once and store the corresponding sequence ids sequentially, as shown in Fig. 1 d. After the index merging, we only need a small array for the sorted subject offsets. Furthermore, because the index is sorted by subject offsets, instead of storing the absolute value of subject offsets, we store the incremental subject offsets, as noted in Fig. 1 e, and only use eight (8) bits for the incremental subject offsets. Because the number of positions for a specific subject offset in one block is generally less than 256, we can also use eight (8) bits for the number of positions. Thus, in total, we only need a 16-bit integer to store a subject offset and its number of positions.

**Fig. 2:**
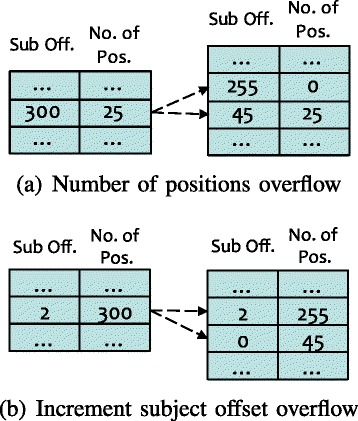
An example of resolving overflows in the compressed index. **a** Resolving the overflow in the number of positions. **b** Resolving in the incremental subject offsets

However, this compressed method presents a challenge. When we use eight (8) bits each for the incremental subject offset and the number of repeated positions, there still exist a few cases that the increment subject offsets or the number of repeated positions is larger than 255. When such situations are encountered, we split one position entry into multiple entries to make the value less than 255. For example, as shown in Fig. 2 a, if the increment subject offset is 300 with 25 positions, then we split the subject offset into two entries, where the first entry has the incremental subject offset 255 and the number of repeated position 0, and the second entry has the incremental subject offset 45 for the 25 positions. Similarly, as shown in Fig. 2 b, for 300 repeated number of positions, the subject offset is split into two entries, where the first entry has the incremental subject offset 2 for 255 positions, but the second has the incremental subject offset 0 for an additional 45 positions.

### Optimized BLASTP algorithm with database index

Because the BLASTP search algorithm introduces a more irregular memory access pattern when using a database index (rather than a query index), we propose and realize *hit reordering with two-level binning* in order to mitigate the irregular memory access pattern and irregular control flow, especially for the two-hit ungapped extension.

#### Hit reordering with two-level binning

The two-hit ungapped extension in protein sequence search requires searching for two-hit pairs, where two hits are on the same diagonal and close together, to trigger ungapped extensions. The traditional method, namely the *last-hit array-based method*, is commonly used in query-indexed BLAST. The last-hit array method uses an array to record the last hit of each diagonal. When a new hit is detected, the algorithm checks the distance between the newly found hit and the last hit in the same diagonal of the last-hit array and updates the last hit with the new hit. Although the algorithm scans the subject sequence from the beginning to the end, the diagonal access for a new hit can be random. The random memory accesses on last-hit arrays is a critical problem for database-indexed BLAST, which aligns a query to thousands of subject sequences at once (rather than aligning a subject sequence to a single query, as is done in query-indexed BLAST). Therefore, to improve the performance of finding two-hit pairs, we propose a new method that reorders hits with two-level binning.

**Fig. 3:**
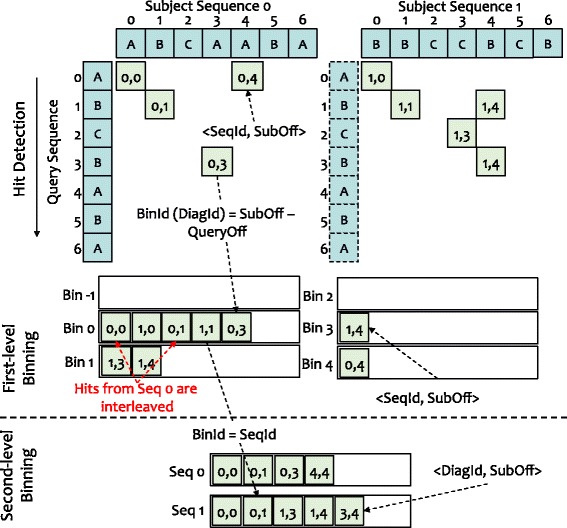
An example of two-level binning *without* filtering. First-level binning groups hits into bins according to their diagonal ids. Second-level binning scans hits in the first-level bins bin by bin, and regroups hits into second-level bins by their sequence ids

As shown in Fig. 3, each bin is mapped to a diagonal in the first level of binning, and the hits are grouped into bins by diagonal ids, which are calculated by subject offsets minus query offsets. Because query offsets can be calculated by subject offsets minus diagonal ids, we only store the sequence ids and subject offsets directly from the index in order to to minimize memory usage.

**Fig. 4:**
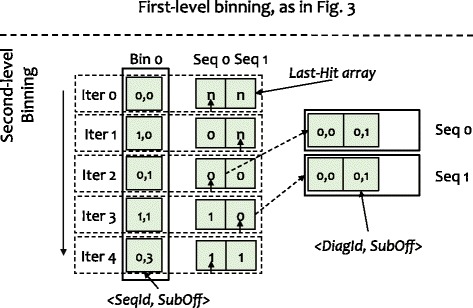
An example of two-level binning *with* filtering. While scanning hits in first-level bins, we check the distance of each hit to the last hit in the last-hit array. Only if the distance fits into the threshold, the hit can be put into the second-level bins

After the first-level binning, hits having the same diagonal ids are placed into the same bins. However, in each bin, the hits from different sequences are interleaved. Thus, we design a second level of binning to reorder the hits by sequence ids. In contrast to first-level binning, where the bin id is equal to the diagonal id, second-level binning sets the bin id to the sequence id. Because we scan the bins of the first-level binning one by one, the hits in a second-level bin are sorted naturally by the diagonal id. As shown in Fig. 4, a hit in the second-level bin contains the subject offset and the diagonal id. With the second-level binning, hits from different sequences are put into different bins and sorted by diagonal ids. After that, we can quickly detect two-hit pairs by scanning every second-level bin.

To improve the performance of the two-hit ungapped extension further, we filter out the hits that cannot be used to trigger the ungapped extension (instead of directly putting all the hits into the second-level bins). This optimization, as captured in Fig. 4, can dramatically reduce processing overhead by reducing memory usage, and in turn, improving performance.

Specifically, before writing a hit into a second-level bin, we check its distance to the last hit in last-hit array. Only if the distance of the current hit to the last hit satisfies the distance thresholds, i.e., less than *threshold*_*A* and greater than or equal to *overlap*, the hit can be put into the second-level bins. As the number of sequences in a index block can be adjusted by configuring the size of the index block, the size of the last-hit array may be small enough to fit in the cache: not only in the last-level cache (LLC) on the Haswell CPU in our evaluation but also in the L2 cache. As a result, this optimization to ungapped extension exhibits excellent data locality when accessing the reordered hits, thus improving performance. Moreover, because our optimization filters out the majority of hits, we also significantly reduce the time spent on memory-write operations, and in turn, improve performance further.

**Fig. 5:**
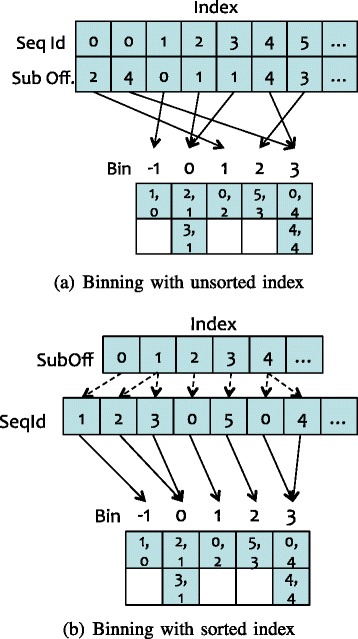
An example of first-level binning hits with unsorted index and sorted index. In the example, the hits are generated for the word in query offset 1. **a** First-level binning with unsorted index. **b** First-level binning with sorted index

If the subject offsets are unsorted in the database index, as shown in Fig. 5 a, the binning method can introduce random memory accesses, which would adversely impact performance. However, sorting the subject offsets in the database index, as shown in in Fig. 1 c, can resolve this problem. Once the index sorting is complete, as shown in Fig. 5 b, both the reads on the database index and the writes on the first-level binning are contiguous, thus improving the binning performance via better data locality.

### Optimizations via multithreading

In BLAST algorithm, the query sequence is aligned to each subject sequence in the database independently and iteratively. Thus, we can parallelize the BLAST algorithm with OpenMP multithreading on the multicore processors in a compute node, e.g., our pair of 12-core Intel Haswell CPUs or 24 cores in total. However, achieving robust scalability on such multicore processors is non-trivial, particularly for a data-/memory-intensive program like BLAST, which also introduces irregular memory access patterns as well as irregular control flow. At a high level, two major challenges exist for parallelizing BLAST within a compute node: (1) cache and memory contention among threads on different cores and (2) load balancing among these threads.

Because the alignment on each query is independent, a straightforward approach to parallelization maps the alignment of each query to a thread. However, this approach results in different threads potentially accessing different index blocks at the same time. In light of the limited cache size, this approach results in severe cache contention between threads. To mitigate this cache contention and maximize cache-sharing across threads, we exchange execution order, as shown in Algorithm 1. That is, the first two stages, i.e., hit detection and ungapped extension, which share the same database index, access the same database block for all batch query sequences (from Line 6 to 10). So, we apply the OpenMP pragma on the inner loop to make different threads process *different* input query sequences but on the *same* index block. Then, threads on different cores may share the database index that is loaded into memory and even cache. The aligned results for each index block are then merged together for the final alignment with traceback, as shown on Line 9.



For better load balancing, and in turn, better performance, we leverage the fact that we already have a sorted database with respect to sequence lengths. We then partition this database into blocks of equal size and leverage OpenMP dynamic scheduling.

### Discussion

In muBLASTP, we use the composition-based statistics presented in [27], which is also the default method used in NCBI BLAST. For other composition-based statistics methods in NCBI BLAST, such as [28], our current code base does not support it. We leave this work for the future versions.

Moreover, the current version of muBLASTP can only produce the identical results to NCBI BLAST when both use the default output format (i.e., “pairwise” format) and the default composition-based statistics method. As a result, our software can only generate the similar results to NCBI BLAST if any other parameter is set. In the future updates of this software, we will add the supports for different formats, making muBLASTP to be a comprehensive tool as NCBI BLAST.

## Results

We conducted our experimental evaluations on two different multicore CPU platforms — Haswell platform and Nehalem platform. Haswell platform consists of two Intel Haswell Xeon CPUs (E5-2680v3), each of which has 12 cores, 30 MB shared L3 cache, and 32 KB L1 cache and 256 KB L2 private cache on each core. Haswell platform also has 128 GB of 2133-MHz DDR main memory. Nehalem platform consists of two Intel Nehalem Xeon CPUs (E5645), each of which has 6 cores, 12 MB shared L3 cache, and 32 KB L1 cache and 256 KB L2 private cache on each core. Nehalem platform also has 24 GB of 1600-MHz DDR main memory. In the experiments, all programs are compiled by an Intel C/C++ compiler 15.3 with the compiler option -O3 -fopenmp. In the experiments, all performance numbers are average values of multiple runs.

**Databases** We used three typical protein NCBI databases from GenBank [29]: *uniprot_sprot*, *env_nr* and *nr*. The *uniprot_sprot* database includes approximately 300,000 sequences with a total size of 250 MB and whose median length and average length are 292 and 355 amino acids (or letters), respectively. The *env_nr* database consists of about 6,000,000 sequences with the total size at 1.7 GB and whose median length and average length are 177 and 197 amino acids (or letters), respectively. The *nr* database consists of about 85,000,000 sequences with the total size at 53 GB and whose median length and average length are 292 and 366 amino acids (or letters), respectively.

**Fig. 6:**
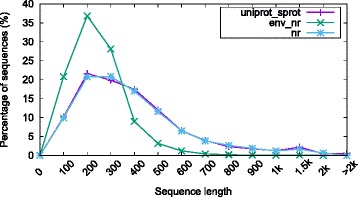
Sequence length distribution of *uniprot_sprot*, *env_nr* and *nr* database

Figure 6 shows the distribution of sequence lengths for the *uniprot_sprot*, *env_nr* and *nr* databases. The sizes of most sequences from the two databases lie in the range from 60 amino acids to 1000 amino acids and with only a handful of sequences longer than 1000 amino acids. Similar observations are also reported in other studies [17, 30, 31].

**Queries** The performance of BLAST depends in part on the query length. Based on the length distribution shown in Fig. 6, we evaluated the performance of our single-thread muBLASTP using three sets of queries with different lengths — around 100, 500 and 1000 — where each query set contains 50 queries. For the evaluation of our multithreaded muBLASTP, we built three query batches, each containing 100 queries with lengths around 100, 500, and 1000, respectively. In addition, we constructed a mixed-length batch of sequences by randomly selecting 100 queries of arbitrary size in order to evaluate the real world performance of multithreaded muBLASTP, especially with respect to scalability and load balancing. Table 1 captures the statistical profile of query lengths from our mixed-length query batches of the *uniprot_sprot*, *env_nr* and *nr* databases, respectively. The details for queries are given in Additional file 1.

**Table 1:** Statistics of query lengths (amino acids) in mixed-length query batches

Target database	Average length	Median length	Maximum length
*uniprot_sprot*	333	289	1187
*env_nr*	191	175	504
*nr*	312	263	1127

To align queries with muBLASTP, as the following commands, we first formatted and sorted the database using the formatdb and sortdb program, respectively. And then, we indexed the database with a configurable block size using the indexdb program, and finally aligned queries against the database using the mublastp program.

- formatdb <–i database>
- sortdb <–i database> <–o sorted_database>
- indexdb <–i sorted_database> ∖
- [–s block_size(K letters), default 128]
- mublastp <–i query> <–d sorted_database> ∖
- [–t number_of_threads]

In experiments, we compared muBLASTP with NCBI BLAST (version 2.3.0), which was configured and built with the following commands.

- ./configure CC=icc CXX=icpc ∖
—without–gui —without–debug
- make
- make install

We formatted database, and ran NCBI BLAST with default parameters, as noted below.

- makeblastdb <–in database> <–dbtype prot>
- blastp <–query query> <–db database> ∖
- [–num_threads number_of_threads]

As the usage of indexdb program shown above, the index block size is a configurable variable. By default, its value is set to 128 K amino acids (or letters), making the index block size around 256 KB and fitting into the L2 cache (256 KB on both Haswell and Nehalem). The reason to set the index block size based on the L2 cache is that since the L2 cache is private for each core, we could avoid heavy cache contentions across different threads in the multithreading mode if the index data can be located from the L2 cache. If muBLASTP is running with a single thread, we could increase the index block size and try to fully utilize the L3 cache as well as the L2 cache. Because increasing index block size may generate much more hits in each block, the practical values are 2048 K amino acids (letters) on Haswell and 1024 K amino acids on Nehalem in our experiments for the single thread mode.

### Index size

Table 2 shows the raw file (FASTA format) size for the corresponding database (“Database” row), the corresponding index file size with neighboring words (“Index w/ neighbors” row), the index file size without neighboring words (“Index w/o neighbors” row), and the compressed index file size (“Compressed index” row). Except “Database” row, the latter three refer to the different indexing mechanisms presented in this paper. According to Table 2, the database index *with* neighboring words, when compared to the database index *without* neighboring words, can be on the order of 20 times larger. Index compression achieves 1.47-fold compression rate for the *uniprot_sprot* database, 1.46-fold compression rate for the *env_nr* database, and 1.47-fold compression rate for the *nr* database. As a result, the compressed (database) index for the *uniprot_sprot* database is 2 times the size of the original database while it is 1.8 times the size of the original *env_nr* database, and it is 1.6 times the size of the original *nr* database. Because we embedded the offsets to neighboring words into the database index, our index without neighboring words can achieve identical results as the index with neighboring words but with significantly less memory usage.

**Table 2:** Size of database and index files in gigabytes (GB)

	*uniprot_sprot*	*env_nr*	*nr*
Database	0.25	1.89	52.4
Index w/ neighbors	18.1	116.6	N/A
Index w/o neighbors	0.76	5.82	122.3
Compressed index	0.51	3.97	83.1

### Performance comparison for alignment stages

To evaluate the performance improvement with index structure and re-factored BLAST algorithm, we used gettimeofday() functions to measure the execution time of all four alignment stages for both muBLASTP and NCBI BLAST without I/O.

#### Single-threaded muBLASTP vs. single-threaded NCBI BLAST

**Fig. 7:**
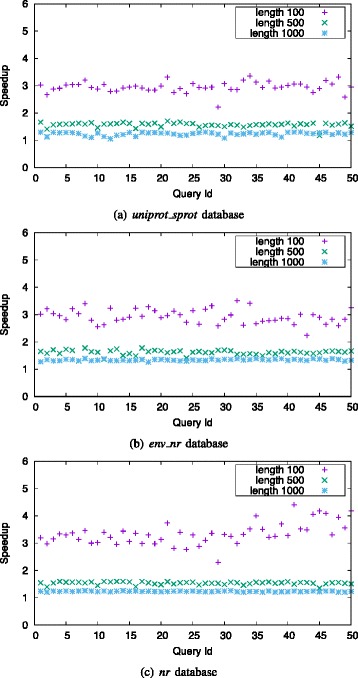
Speedup for alignment stages of single-threaded muBLASTP over single-threaded NCBI BLAST on Haswell platform with different query lengths on *uniprot_sprot* database (**a**), *env_nr* database (**b**) and *nr* database (**c**)

Figure 7 shows the speedups of singled-threaded muBLASTP over single-threaded NCBI BLAST on Haswell platform, using different query lengths. muBLASTP achieves 2.22 ∼3.35-fold, 1.17 ∼1.7-fold, and 1.06 ∼1.3-fold speedups over NCBI BLAST on the *uniprot_sprot* database with queries of length 100, 500, and 1000, respectively. For the *env_nr* database, muBLASTP achieves 2.24 ∼3.51-fold, 1.3 ∼1.77-fold, and 1.26 ∼1.39-fold speedups with queries of length 100, 500 and 1000, respectively. For the *nr* database, muBLASTP achieves 2.3 ∼4.41-fold, 1.34 ∼1.5-fold, and 1.21 ∼1.26-fold speedups with queries of length 100, 500 and 1000, respectively. muBLASTP achieves higher speedup on the larger database because the BLAST algorithm on a large database needs to process significantly more hits, i.e., spending more time on hit detection and two-pair hit ungapped extension, which are the stages that our optimizations focus on.

**Fig. 8:**
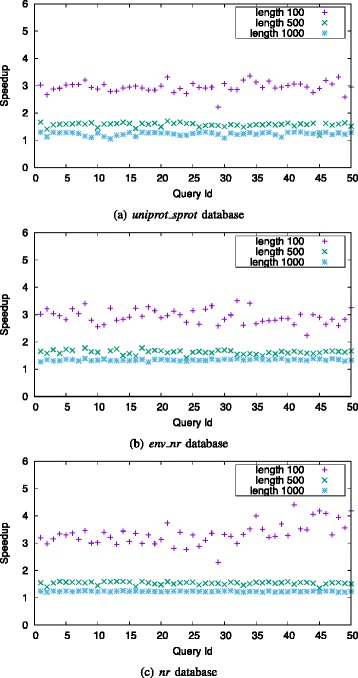
Speedup for alignment stages of single-threaded muBLASTP over single-threaded NCBI BLAST on Nehalem platform with different query lengths on *uniprot_sprot* database (**a**), *env_nr* database (**b**) and *nr* database (**c**)

Figure 8 shows the speedups of singled-threaded muBLASTP over single-threaded NCBI BLAST on Nehalem platform, using different query lengths. muBLASTP achieves 1.6 ∼3.17-fold, 1.33 ∼1.47-fold, and 1.09 ∼1.3-fold speedups over NCBI BLAST on the *uniprot_sprot* database with queries of length 100, 500, and 1000, respectively. For the *env_nr* database, muBLASTP achieves 2.38 ∼3.8-fold, 1.3 ∼1.53-fold, and 1.14 ∼1.25-fold speedups with queries of length 100, 500 and 1000, respectively. For the *nr* database, muBLASTP achieves 2.0 ∼3.21-fold, 1.1 ∼1.49-fold, and 1.00 ∼1.25-fold speedups with queries of length 100, 500 and 1000, respectively.

#### Multithreaded muBLASTP vs. multithreaded NCBI BLAST

When using query batches of different lengths, Table 3 shows that our multithreaded muBLASTP on Haswell platform achieves up to a 5.7-fold speedup over multithreaded NCBI BLAST when using the *uniprot_sprot* database, up to a 2.67-fold speedup when using the *env_nr* database, and up to a 1.94-fold speedup when using the *nr* database.

**Table 3:** Speedup for alignment stages of multithreaded muBLASTP over multithreaded NCBI BLAST on Haswell platform (with 24 threads) and Nehalem platform (with 12 threads) with query batches of different query length

Query length	Haswell platform						Nehalem platform					
	*uniprot_sprot*		*env_nr*		*nr*		*uniprot_sprot*		*env_nr*		*nr*	
	Aver	SD	Aver	SD	Aver	SD	Aver	SD	Aver	SD	Aver	SD
100	5.7	0.013	2.67	0.013	1.94	0.021	8.2	0.013	4.52	0.017	8.59	0.015
500	3.22	0.012	1.44	0.012	1.39	0.014	2.25	0.018	1.69	0.018	2.69	0.016
1000	2.85	0.016	1.35	0.013	1.20	0.015	1.76	0.018	1.41	0.016	1.72	0.015
Mixed	4.16	0.013	1.41	0.012	1.49	0.012	2.82	0.018	2.34	0.013	4.1	0.015

*Aver* is the average value of three runs, and *SD* is the standard deviation of three runs

Table 3 shows that our multithreaded muBLASTP on Nehalem platform achieves up to a 8.2-fold speedup over multithreaded NCBI BLAST when using the *uniprot_sprot* database, up to a 4.52-fold speedup when using the *env_nr* database, and up to a 8.59-fold speedup when using the *nr* database. In this case, muBLASTP achieves much higher speedups on the smaller *uniprot_sprot* database, which indicates that muBLASTP delivers better scalability than NCBI BLAST on smaller databases.

We also tested muBLASTP performance with query batches of mixed lengths. Table 3 shows that on Haswell platform muBLASTP achieves a 4.16-fold speedup over NCBI BLAST on *uniprot_sprot* database, a 1.41-fold speedup over NCBI BLAST on *env_nr* database, and a 1.49-fold speedup on *nr* database. Table 3 also shows that on Nehalem platform muBLASTP achieves a 2.82-fold speedup over NCBI BLAST on *uniprot_sprot* database, a 2.34-fold speedup over NCBI BLAST on *env_nr* database, and a 4.1-fold speedup on *nr* database.

#### Multithreaded muBLASTP vs. single-threaded muBLASTP

We also evaluated parallel efficiency of multithreaded muBLASTP. Table 4 shows that multithreaded muBLASTP using 24 threads on Haswell platform can achieve 19.8 ∼21.6-fold speedups over single-thread muBLASTP with query batches of different lengths on different databases. Table 4 also shows that multithreaded muBLASTP using 12 threads on Nehalem platform can achieve 10.7 ∼11.6-fold speedups over single-thread muBLASTP with query batches of different lengths on different databases.

**Table 4:** Speedup for alignment stages of multithreaded muBLASTP over single-threaded muBLASTP on Haswell platform (with 24 threads) and Nehalem platform (with 12 threads) with query batches of different query length

Query length	Haswell platform						Nehalem platform					
	*uniprot_sprot*		*env_nr*		*nr*		*uniprot_sprot*		*env_nr*		*nr*	
	Aver	SD	Aver	SD	Aver	SD	Aver	SD	Aver	SD	Aver	SD
100	19.8	0.013	20.2	0.016	20.0	0.013	10.7	0.014	11.2	0.012	11.6	0.012
500	20.9	0.022	20.6	0.011	21.4	0.017	10.9	0.012	11.5	0.013	11.5	0.012
1000	21.4	0.013	21.2	0.013	21.5	0.012	10.8	0.012	11.3	0.013	11.6	0.018
Mixed	21.4	0.012	21.5	0.017	21.6	0.013	10.9	0.018	11.3	0.018	11.5	0.015

*Aver* is the average value of three runs, and *SD* is the standard deviation of three runs

### End-to-end performance comparison

To evaluate the end-to-end performance of muBLASTP, we measured the end-to-end execution time of the program via Linux *time* command. To minimize the impacts disk I/O, we loaded database and index into RAM disk, i.e., tmpfs, which is a memory based file system for fast and stable disk I/O.

#### Single-threaded muBLASTP vs. single-threaded NCBI BLAST

**Fig. 9:**
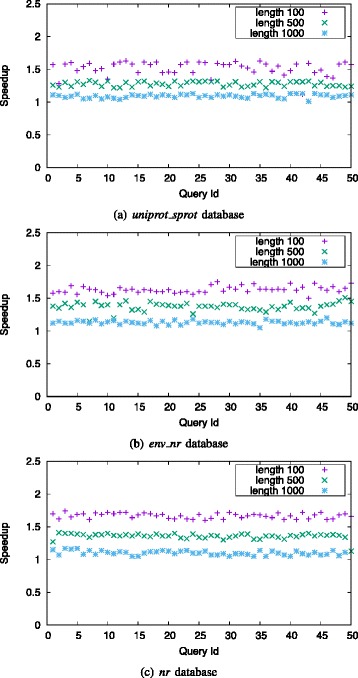
End-to-end speedup of single-threaded muBLASTP over single-threaded NCBI BLAST on Haswell platform with different query lengths on *uniprot_sprot* database (**a**), *env_nr* database (**b**) and *nr* database (**c**)

Figure 9 shows the speedups of singled-threaded muBLASTP over single-threaded NCBI BLAST on Haswell platform, using different query lengths. muBLASTP achieves 1.12 ∼1.63-fold, 1.22 ∼1.33-fold, and 1.01 ∼1.13-fold speedups over NCBI BLAST on the *uniprot_sprot* database with queries of length 100, 500, and 1000, respectively. For the *env_nr* database, muBLASTP achieves 1.5 ∼1.75-fold, 1.05 ∼1.2-fold, and 1.26 ∼1.39-fold speedups with queries of length 100, 500 and 1000, respectively. For the *nr* database, muBLASTP achieves 1.6 ∼1.74-fold, 1.27 ∼1.41-fold, and 1.05 ∼1.17-fold speedups with queries of length 100, 500 and 1000, respectively.

**Fig. 10:**
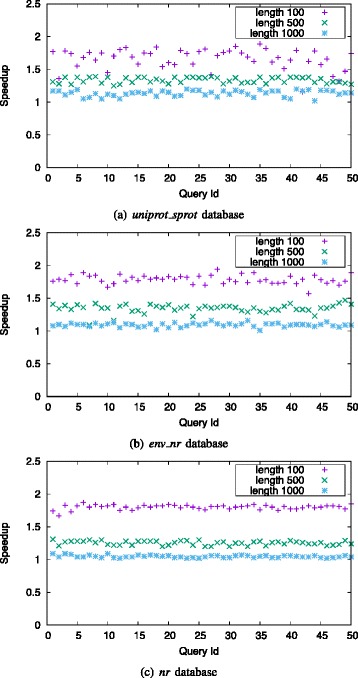
End-to-end speedup of single-threaded muBLASTP over single-threaded NCBI BLAST on Nehalem platform with different query lengths on *uniprot_sprot* database (**a**), *env_nr* database (**b**) and *nr* database (**c**)

Figure 10 shows the speedups of singled-threaded muBLASTP over single-threaded NCBI BLAST on Nehalem platform, using different query lengths. muBLASTP achieves 1.16 ∼1.89-fold, 1.25 ∼1.39-fold, and 1.02 ∼1.2-fold speedups over NCBI BLAST on the *uniprot_sprot* database with queries of length 100, 500, and 1000, respectively. For the *env_nr* database, muBLASTP achieves 1.57 ∼1.94-fold, 1.09 ∼1.47-fold, and 1.01 ∼1.16-fold speedups with queries of length 100, 500 and 1000, respectively. For the *nr* database, muBLASTP achieves 1.67 ∼1.87-fold, 1.2 ∼1.31-fold, and 1.02 ∼1.09-fold speedups with queries of length 100, 500 and 1000, respectively.

#### Multithreaded muBLASTP vs. multithreaded NCBI BLAST

When using query batches of different lengths, Table 5 shows that our multithreaded muBLASTP on Haswell platform achieves up to a 4.56-fold speedup over multithreaded NCBI BLAST when using the *uniprot_sprot* database, up to a 2.62-fold speedup when using the *env_nr* database, and up to a 1.81-fold speedup when using the *nr* database.

**Table 5:** End-to-end speedup of multithreaded muBLASTP over multithreaded NCBI BLAST on Haswell platform (with 24 threads) and Nehalem platform (with 12 threads) with query batches of different query length

Query length	Haswell platform						Nehalem platform					
	*uniprot_sprot*		*env_nr*		*nr*		*uniprot_sprot*		*env_nr*		*nr*	
	Aver	SD	Aver	SD	Aver	SD	Aver	SD	Aver	SD	Aver	SD
100	4.56	0.002	2.62	0.008	1.81	0.010	3.85	0.001	2.2	0.002	1.56	0.001
500	2.45	0.005	1.41	0.002	1.35	0.002	1.82	0.002	1.34	0.001	1.17	0.002
1000	2.74	0.003	1.32	0.003	1.19	0.001	1.71	0.002	1.13	0.001	1.05	0.002
Mixed	3.28	0.005	1.38	0.002	1.47	0.002	2.61	0.001	1.61	0.002	1.24	0.002

*Aver* is the average value of three runs, and *SD* is the standard deviation of three runs

Table 5 shows that our multithreaded muBLASTP on Nehalem platform achieves up to a 3.85-fold speedup over multithreaded NCBI BLAST when using the *uniprot_sprot* database, up to a 2.2-fold speedup when using the *env_nr* database, and up to a 1.56-fold speedup when using the *nr* database.

We also tested muBLASTP performance with query batches of mixed lengths. Table 5 shows that on Haswell platform muBLASTP achieves a 3.28-fold speedup over NCBI BLAST on *uniprot_sprot* database, 1.38-fold speedup over NCBI BLAST on *env_nr* database, and a 1.47-fold speedup on *nr* database. Table 5 shows that on Nehalem platform muBLASTP achieves a 2.61-fold speedup over NCBI BLAST on *uniprot_sprot* database, a 1.61-fold speedup over NCBI BLAST on *env_nr* database, and a 1.24-fold speedup on *nr* database.

#### Multithreaded muBLASTP vs. single-threaded muBLASTP

We also evaluated parallel efficiency of multithreaded muBLASTP with end-to-end execution time. Table 6 shows that multithreaded muBLASTP using 24 threads on Haswell platform can achieve up to a 20.5-fold speedup over single-thread muBLASTP with query batches of different lengths on different databases. Table 6 also shows that multithreaded muBLASTP using 12 threads on Nehalem platform can achieve up to a 11.1-fold speedup over single-thread muBLASTP with query batches of different lengths on different databases.

**Table 6:** End-to-end speedup of multithreaded muBLASTP over single-threaded muBLASTP on Haswell platform (with 24 threads) and Nehalem platform (with 12 threads) with query batches of different query length

Query length	Haswell platform						Nehalem platform					
	*uniprot_sprot*		*env_nr*		*nr*		*uniprot_sprot*		*env_nr*		*nr*	
	Aver	SD	Aver	SD	Aver	SD	Aver	SD	Aver	SD	Aver	SD
100	16.2	0.001	16.8	0.002	17.3	0.003	9.2	0.001	9.9	0.001	6.3	0.003
500	20.4	0.002	20.3	0.002	20.5	0.002	11.1	0.004	10.6	0.003	11.1	0.002
1000	19.4	0.002	19.3	0.001	19.6	0.004	10.7	0.002	10.2	0.002	10.9	0.001
Mixed	19.2	0.001	19.1	0.002	19.3	0.003	10.3	0.002	11.1	0.002	10.4	0.002

*Aver* is the average value of three runs, and *SD* is the standard deviation of three runs

## Conclusions

In this paper, we present muBLASTP, a database-indexed BLASTP that delivers identical hits returned to NCBI BLAST for protein sequence search. With our new index structure for protein databases and associated optimizations in muBLASTP, we deliver a re-factored BLASTP algorithm for modern multicore processors that achieves much higher throughput with acceptable memory usage for the database index. Those optimizations and techniques in index structure and BLAST algorithm, such as index compression, sorting index, two-level binning, etc., are not merely beneficial to database-indexed search for protein sequence, also can be propagated to nucleotide sequence search and other alignment algorithms.

On a modern compute node with a total of 24 Intel Haswell CPU cores, the multithreaded muBLASTP achieves up to a 5.7-fold speedup for alignment stages, and up to a 4.56-fold end-to-end speedup over multithreaded NCBI BLAST. muBLASTP also can achieve significant speedups on an older generation platform with dual 6 cores Intel Nehalem CPU, where muBLASTP delivers up to a 8.59-fold speedups for alignment stages, and up to a 3.85-fold end-to-end speedup over multithreaded NCBI BLAST.

In the future, we plan to extend muBLASTP to many-core architectures, e.g., Intel Xeon Phi, which currently contains 60 cores and supports 240 threads. The more complex cache/memory hierarchy may lead to significant challenges in achieving high throughput for the multithreaded BLAST algorithm. In addition, we plan to integrate our database-indexed BLASTP into mpiBLAST, thus combining intra-node and inter-node parallelism for even greater performance benefit on a high-performance computing cluster.

## Availability and requirements

- **Project name:** muBLASTP
- **Project home page:** https://github.com/vtsynergy/muBLASTP
- **Operating system(s):** UNIX / Linux
- **Programming language:** C/C++
- **License:** LGPL v2.1

## Additional file

Additional file 1 Tables of information for queries in the experiments. For each database, there are four tables for queries of length 100, 500, 1000 and mixed. Each table contains 100 queries in total for performance comparison of multithreading, and first 50 queries for single-threaded performance comparison. (PDF 75.2 kb)

## Abbreviations

BLAST: Basic local alignment search tool
CPU: Central processing unit
DFA: Deterministic finite automaton
FPGA: Field-programmable gate array
GPU: Graphics processing unit
NCBI: National center for biotechnology information
NGS: Next-generation sequencing
