# Supplementary material for: muBLASTP: database-indexed protein sequence search on multicore CPUs
Source: BMC Bioinformatics. 2016 Nov 4;17:443. doi: 10.1186/s12859-016-1302-4 (PMC5096327; doi:10.1186/s12859-016-1302-4)
Supplement: Additional file 1 — Tables of information for queries in the experiments. For each database, there are four tables for queries of length 100, 500, 1000 and mixed. Each table contains 100 queries in total for performance comparison of multithreading, and first 50 queries for single-threaded performance comparison. (PDF 75.2 kb) [file 12859_2016_1302_MOESM1_ESM.pdf]

muBLASTP: Database-Indexed Protein Sequence Search on Multicore CPUs — Additional File

Jing Zhang*, Sanchit Misra, Hao Wang and Wu-chun Feng

Table 1 Queries of length 100 for uniprot_sprot database

Id	Accession No.	Length	Id	Accession No.	Length
1	Q91G65	100	51	P24301	100
2	Q66125	100	52	B2HD09	100
3	P80353	100	53	P60533	100
4	O60516	100	54	A0QSS3	100
5	P20194	100	55	A5U893	100
6	P12820	100	56	P9WPE4	100
7	Q72CU1	100	57	P9WPE5	100
8	A1VEL6	100	58	A0PME8	100
9	Q5YS19	100	59	A1T576	100
10	A5GM25	100	60	Q5Z1G0	100
11	P81644	100	61	Q9XCB0	100
12	E2RAK7	100	62	P08929	100
13	P0DJG2	100	63	P37854	100
14	P02652	100	64	P37852	100
15	P0DM93	100	65	P07231	100
16	P18656	100	66	A4QGR5	100
17	P0DM94	100	67	Q8NMQ1	100
18	P0DM95	100	68	B8DJU4	100
19	P0DM96	100	69	Q5F9I5	100
20	Q8MIQ5	100	70	Q9JZZ5	100
21	P27916	100	71	P0C8B3	100
22	P0DMN9	100	72	Q9BXV9	100
23	P12279	100	73	P0C8B5	100
24	P0DMP9	100	74	Q6UWE3	100
25	Q7Z4Y8	100	75	Q2XXR4	100
26	O66564	100	76	Q556L8	100
27	A7ZC73	100	77	P10889	100
28	A7GX83	100	78	P30348	100
29	A0RQF4	100	79	Q6W5C0	100
30	P0C6P0	100	80	A9QWQ1	100
31	Q6FY24	100	81	Q9JHH5	100
32	P84291	100	82	P29669	100
33	P00974	100	83	P00087	100
34	P04815	100	84	Q89AA6	100
35	P33719	100	85	P64387	100
36	Q8YQ90	100	86	Q9Z8C7	100
37	P0CE67	100	87	P64386	100
38	Q5RHZ2	100	88	P73418	100
39	Q9TTQ4	100	89	Q01524	100
40	P72759	100	90	Q5G860	100
41	Q06153	100	91	P0C8A8	100
42	A8YXZ5	100	92	Q2V4J8	100
43	Q8N0T1	100	93	Q89681	100
44	A0QKR3	100	94	P0C232	100
45	P60532	100	95	P11547	100
46	P15020	100	96	Q751F9	100
47	A1KPA9	100	97	P40932	100
48	C1AHN1	100	98	B3MQ24	100
49	A4TEN7	100	99	B4H2S0	100
50	B8ZUD1	100	100	Q29IN4	100

Table 2 Queries of length 500 for uniprot_sprot database

Id	Accession No.	Length	Id	Accession No.	Length
1	Q28647	500	51	Q73IU2	500
2	Q93YV6	500	52	Q3IP82	500
3	P24758	500	53	P32629	500
4	A2Z212	500	54	B7GGW0	500
5	Q0J185	500	55	B3PD57	500
6	Q92455	500	56	A8ALP0	500
7	P12390	500	57	A7ZHF3	500
8	Q9UW95	500	58	B7UIA8	500
9	Q3Z7F6	500	59	B7MAI5	500
10	P15437	500	60	B7L4I3	500
11	P86886	500	61	P58538	500
12	P12762	500	62	B5YZ98	500
13	P81178	500	63	B7NHG8	500
14	P54115	500	64	B7MNR9	500
15	O74187	500	65	B7M0F7	500
16	Q7ZW24	500	66	C4ZPY5	500
17	Q8EI85	500	67	A7ZW12	500
18	B7J5I9	500	68	A1A7A9	500
19	B5EKZ2	500	69	Q0TLS8	500
20	A6TWW9	500	70	Q8FL89	500
21	Q5NXM1	500	71	B1IRB6	500
22	A1K9L5	500	72	P08202	500
23	O32106	500	73	B7N7T6	500
24	Q1LTH8	500	74	B6HZ42	500
25	Q6MH10	500	75	B1LFZ6	500
26	Q5L681	500	76	Q1RGD7	500
27	Q254B9	500	77	A4W6G6	500
28	Q6NG90	500	78	B7LVT3	500
29	Q8NNJ4	500	79	B5Y1Y1	500
30	Q5FFZ5	500	80	A6T4K0	500
31	Q5HAP2	500	81	Q1IUW9	500
32	Q1QKW6	500	82	Q21MP3	500
33	Q3SS04	500	83	B5F783	500
34	B6JGL8	500	84	A9MQF1	500
35	Q2IX74	500	85	Q57TF9	500
36	Q6N5B9	500	86	B5FI45	500
37	Q135P8	500	87	B5R1T9	500
38	B3Q9R8	500	88	B5RGD3	500
39	C3PMI0	500	89	B4TJ59	500
40	A8EXL1	500	90	B4SU23	500
41	Q92J85	500	91	Q5PDF2	500
42	Q4UKD7	500	92	A9MYN8	500
43	A8F0Q0	500	93	B5BL43	500
44	P27888	500	94	B4TWU8	500
45	C4K1N6	500	95	P58539	500
46	B0BWB2	500	96	P06189	500
47	A8GQW7	500	97	B8EEN5	500
48	Q68XM6	500	98	A0KWX7	500
49	Q21KZ5	500	99	Q0HIR5	500
50	C4LA51	500	100	Q0HV71	500

*Correspondence: zjing14@vt.edu
Full list of author information is available at the end of the article

Table 3 Queries of length 1000 for uniprot_sprot database

Id	Accession No.	Length	Id	Accession No.	Length
1	C8V212	1000	51	Q6NU21	1001
2	Q86YS7	1000	52	Q7ZYJ0	1001
3	Q5RDC8	1000	53	Q7L7X3	1001
4	P27895	1000	54	Q5F2E8	1001
5	Q1ZXJ0	1000	55	O88664	1001
6	Q20168	1000	56	Q13472	1001
7	Q6NU40	1000	57	A3PWJ3	1001
8	Q8C4S8	1000	58	Q9Y824	1001
9	Q81ZW1	1000	59	Q8CFW1	1002
10	Q9W1M8	1000	60	Q292Q0	1002
11	Q9Y802	1000	61	Q9M2Z1	1002
12	Q6CPV1	1000	62	Q8CJB9	1002
13	O74945	1000	63	O13849	1002
14	Q9Y6Y8	1000	64	Q96JQ2	1002
15	Q86DA5	1000	65	Q07497	1002
16	Q6CIT4	1000	66	Q3TYG6	1002
17	P34619	1000	67	Q08379	1002
18	B4MR46	1000	68	Q91VB4	1002
19	Q5ZKA2	1000	69	Q5ZLK7	1002
20	Q6BL58	1000	70	P39730	1002
21	Q55445	1000	71	Q71S21	1002
22	Q52149	1000	72	A2YQ56	1002
23	Q9FCI4	1000	73	Q69UZ3	1002
24	Q8N283	1001	74	Q8VDG6	1002
25	O14983	1001	75	A1A278	1002
26	P04191	1001	76	P65373	1002
27	O75150	1001	77	P9WJU0	1002
28	Q4R7K7	1001	78	P9WJU1	1002
29	Q3U319	1001	79	Q6CQX2	1002
30	Q5RAU7	1001	80	Q8VHN2	1002
31	Q7V9U4	1001	81	P31630	1002
32	Q8NEM8	1001	82	Q8L6J5	1002
33	Q8VHK9	1001	83	Q93Y94	1002
34	Q9S7J8	1001	84	P59158	1002
35	Q01W31	1001	85	P55018	1002
36	P72689	1001	86	P43612	1002
37	Q9ZJ57	1001	87	Q8GYD9	1002
38	A6WXF0	1001	88	Q95WA4	1002
39	Q0TR53	1001	89	P48526	1002
40	Q8XL08	1001	90	Q89DF8	1002
41	Q64M78	1001	91	A5F398	1002
42	Q94526	1001	92	P0C6Q7	1002
43	Q9JMC1	1001	93	Q00037	1002
44	Q9MAT0	1001	94	Q8JYK0	1002
45	P80560	1001	95	Q24314	1002
46	Q8IXT5	1001	96	P58612	1002
47	F4IV66	1001	97	P93820	1002
48	Q9R0X5	1001	98	A1T636	1002
49	Q08162	1001	99	P25992	1002
50	Q2VWA4	1001	100	Q9NQ90	1003

Table 4 Queries of mixed length for uniprot_sprot database

Id	Accession No.	Length	Id	Accession No.	Length
1	A6MMM5	31	51	Q8ZLJ4	292
2	P82707	46	52	P16286	297
3	P24780	60	53	Q6PBN2	303
4	Q0ZIZ9	66	54	Q2IW81	308
5	Q2V329	73	55	A2RC40	312
6	A4YT76	79	56	Q1GZG8	317
7	B7N0H8	85	57	P33412	323
8	H1ZZI2	89	58	C1KWW2	329
9	Q7V9Y9	94	59	Q7VKV5	334
10	P66733	99	60	C0R200	339
11	B3EGY8	103	61	B5XZ47	344
12	A7I5P8	108	62	P62723	349
13	A4II29	114	63	Q62AI9	355
14	Q31T86	119	64	B7UQ99	360
15	B2V4E9	122	65	Q29423	366
16	B8F4P3	127	66	P23441	372
17	A0QHC1	131	67	C0Q071	379
18	B1X6G5	136	68	B8CW61	384
19	Q2G214	141	69	A2XSL4	391
20	Q58T60	145	70	P9WMU8	397
21	Q7MN57	149	71	Q6AX53	405
22	Q3YUA7	153	72	P9WJX6	413
23	Q5FZI6	156	73	Q54HY8	421
24	Q2SMN6	160	74	Q88QT4	427
25	P0A4C9	165	75	Q9Z2I8	433
26	Q32D37	171	76	O61764	441
27	O98693	176	77	Q8ZN58	449
28	P16741	180	78	Q3TJD7	457
29	Q8YHM8	185	79	P0C1Z2	466
30	Q6ZR85	190	80	A8AJE9	474
31	P32459	195	81	A1WVR7	484
32	Q0II41	199	82	Q07XN9	493
33	Q9CIS2	203	83	Q7NA81	503
34	P53655	207	84	B9E8Y0	513
35	Q5M3V0	211	85	A3D7J9	526
36	Q9M0L0	216	86	P38137	543
37	B2I9J1	220	87	A0LE49	556
38	P24473	226	88	A9WP05	573
39	A4SY42	230	89	Q8BZ64	593
40	Q83IW8	235	90	Q1HVF2	613
41	Q3BVV3	240	91	A0Q762	634
42	Q971Z6	245	92	Q7VLX8	659
43	Q87LD3	250	93	Q328L9	689
44	Q0AP84	255	94	Q5MZ99	720
45	A1KTD5	259	95	Q9SJE1	760
46	B1HTF0	265	96	Q15MZ1	811
47	A7GTW1	270	97	Q2P497	869
48	B4EBE8	275	98	Q9QWG5	928
49	Q6KIS8	281	99	P18907	1021
50	O28147	286	100	A9BF33	1187

Table 5 Queries of length 100 for env_nr database

Id	Accession No.	Length	Id	Accession No.	Length
1	139615335	100	51	140413579	100
2	140316088	100	52	140006960	100
3	136940263	100	53	136945600	100
4	138510479	100	54	141026926	100
5	142508111	100	55	141490456	100
6	140638036	100	56	139000825	100
7	142569616	100	57	136948961	100
8	141804023	100	58	143265442	100
9	138785064	100	59	139877500	100
10	140767667	100	60	141803743	100
11	136921591	100	61	139623376	100
12	136927268	100	62	141069362	100
13	139621791	100	63	136961885	100
14	138372699	100	64	139750866	100
15	134399509	100	65	139093402	100
16	141196730	100	66	141027510	100
17	140007157	100	67	136965080	100
18	136944069	100	68	139132992	100
19	141804713	100	69	136964010	100
20	139621221	100	70	139625382	100
21	139876961	100	71	136981495	100
22	140004365	100	72	138374001	100
23	136927121	100	73	139345961	100
24	141344156	100	74	136973806	100
25	140765041	100	75	136972421	100
26	136928547	100	76	141198679	100
27	136959850	100	77	141497533	100
28	139129699	100	78	139341871	100
29	139000286	100	79	141501333	100
30	140004922	100	80	142532101	100
31	140639049	100	81	142646847	100
32	136927474	100	82	140900797	100
33	139878225	100	83	141030396	100
34	140639916	100	84	141071346	100
35	142509299	100	85	139753011	100
36	139131239	100	86	138787255	100
37	138371510	100	87	136986363	100
38	134419309	100	88	140640342	100
39	141026311	100	89	134406224	100
40	139749537	100	90	139496456	100
41	136946039	100	91	139347039	100
42	136933491	100	92	140769682	100
43	138785104	100	93	138640754	100
44	136950057	100	94	139345195	100
45	142657699	100	95	136969768	100
46	138636536	100	96	138512421	100
47	140280422	100	97	138199023	100
48	138639003	100	98	139880183	100
49	142791972	100	99	139495726	100
50	139335852	100	100	139753060	100

Table 6 Queries of length 500 for env_nr database

Id	Accession No.	Length	Id	Accession No.	Length
1	142805765	500	51	134788806	500
2	142744135	500	52	134791201	500
3	142693496	500	53	134784848	500
4	142793908	500	54	134804657	500
5	142660330	500	55	134804487	500
6	142818278	500	56	134853787	500
7	142040007	500	57	134861980	500
8	142048558	500	58	134870882	500
9	142537862	500	59	134881167	500
10	142044179	500	60	134875723	500
11	142754900	500	61	134877419	500
12	134329765	500	62	134881904	500
13	134329514	500	63	134889746	500
14	134331440	500	64	134892438	500
15	134340658	500	65	134888404	500
16	134342535	500	66	134900152	500
17	134345888	500	67	134899016	500
18	134360818	500	68	134901416	500
19	134361200	500	69	134902491	500
20	134362380	500	70	134920830	500
21	134364194	500	71	134922117	500
22	134364273	500	72	134933345	500
23	134366729	500	73	134930065	500
24	134367577	500	74	134924455	500
25	134367701	500	75	134932589	500
26	134375858	500	76	134933381	500
27	134382087	500	77	134938870	500
28	134382452	500	78	134946326	500
29	134382639	500	79	134948349	500
30	134383562	500	80	134949502	500
31	134389677	500	81	134945165	500
32	134411863	500	82	134956240	500
33	134492867	500	83	134961862	500
34	134495571	500	84	134958689	500
35	134494641	500	85	134964908	500
36	134559117	500	86	134966928	500
37	134557102	500	87	134967552	500
38	134578936	500	88	134968891	500
39	134633606	500	89	134977906	500
40	134645260	500	90	134980986	500
41	134646985	500	91	134980934	500
42	134680278	500	92	134982174	500
43	134697196	500	93	134983369	500
44	134757284	500	94	134983852	500
45	134756340	500	95	134985543	500
46	134772301	500	96	134987004	500
47	134764481	500	97	134987205	500
48	134774089	500	98	134986572	500
49	134775945	500	99	134988987	500
50	134784801	500	100	134990235	500

Table 7 Queries of length 1000 for env_nr database

Id	Accession No.	Length	Id	Accession No.	Length
1	142012494	1000	51	142429925	1002
2	142017512	1000	52	142591933	1002
3	142031891	1000	53	143069530	1002
4	142043207	1000	54	143094407	1002
5	142179393	1000	55	143733222	1002
6	142525661	1000	56	143846397	1002
7	143310744	1000	57	143923052	1002
8	143515314	1000	58	144150590	1002
9	144169629	1000	59	144161851	1002
10	144191973	1000	60	816619644	1002
11	816698252	1000	61	816619759	1002
12	816708527	1000	62	816619768	1002
13	816729788	1000	63	816619821	1002
14	816731198	1000	64	816676513	1002
15	816778245	1000	65	816691763	1002
16	816817300	1000	66	816732767	1002
17	816825841	1000	67	816748029	1002
18	816826335	1000	68	816766353	1002
19	816827186	1000	69	142041896	1003
20	816835438	1000	70	142022172	1003
21	142019881	1001	71	142128486	1003
22	142020170	1001	72	142176059	1003
23	142048127	1001	73	142205816	1003
24	142086845	1001	74	142410039	1003
25	142098518	1001	75	142579941	1003
26	142140154	1001	76	142719660	1003
27	142202352	1001	77	143064594	1003
28	142368108	1001	78	143165980	1003
29	142438858	1001	79	143272099	1003
30	142643052	1001	80	143359527	1003
31	143114614	1001	81	143999536	1003
32	143559679	1001	82	144025935	1003
33	143640519	1001	83	402664787	1003
34	143816529	1001	84	402688283	1003
35	144190845	1001	85	566276073	1003
36	531023574	1001	86	816746486	1003
37	816760727	1001	87	816751876	1003
38	816619581	1001	88	816767130	1003
39	816619651	1001	89	816826585	1003
40	816702035	1001	90	816838647	1003
41	816714166	1001	91	142553979	1004
42	816734703	1001	92	142835047	1004
43	816769934	1001	93	142901850	1004
44	816797340	1001	94	142947240	1004
45	816807776	1001	95	143163344	1004
46	816832670	1001	96	143207256	1004
47	142616658	1002	97	143341135	1004
48	142040802	1002	98	143569948	1004
49	142148851	1002	99	143878219	1004
50	142177040	1002	100	143880239	1004

Table 8 Queries of mixed length for env_nr database

Id	Accession No.	Length	Id	Accession No.	Length
1	598854877	47	51	134977936	177
2	140705305	60	52	134902228	180
3	141204517	62	53	134840084	183
4	140928694	64	54	134931935	186
5	140516537	66	55	135146690	189
6	140187978	68	56	136236681	192
7	139809410	70	57	137725811	195
8	139787472	72	58	139055769	198
9	140157778	74	59	140937912	201
10	140554256	76	60	142789788	204
11	141162879	78	61	134328296	208
12	142139731	80	62	137470780	211
13	143765982	82	63	141219041	214
14	604359080	84	64	816827795	217
15	135069683	87	65	138064217	221
16	137271205	89	66	142290739	224
17	139226674	91	67	135809514	228
18	141944072	93	68	140636253	231
19	816589676	95	69	134503619	235
20	136362760	98	70	139199851	238
21	139820455	100	71	143745183	241
22	142999362	102	72	137104626	245
23	135371020	105	73	140666281	248
24	139433346	107	74	598811498	251
25	143500432	109	75	137544767	255
26	136171243	112	76	140268927	258
27	140754506	114	77	143281312	261
28	816806333	116	78	135586108	265
29	138723145	119	79	139261929	268
30	406549641	121	80	142862713	271
31	136970711	124	81	137510121	275
32	142136239	126	82	142964267	278
33	135832348	129	83	139382762	282
34	141122975	131	84	136983476	286
35	135318358	134	85	135993248	290
36	141440514	136	86	137156525	294
37	135626672	139	87	141034093	298
38	141244915	141	88	137946867	303
39	135867469	144	89	139744034	308
40	142399569	146	90	137480711	314
41	137330918	149	91	135710091	321
42	594906122	151	92	142101584	329
43	140340446	154	93	816751479	339
44	136581010	157	94	135741269	353
45	604368256	159	95	143931953	368
46	142111917	162	96	816479838	386
47	139559838	165	97	134938263	408
48	137785644	168	98	143120162	433
49	136171000	171	99	143359191	464
50	135545852	174	100	144048086	504

Table 9 Queries of length 100 for nr database

Id	Accession No.	Length	Id	Accession No.	Length
1	489997053	100	51	446170608	100
2	446539296	100	52	489496644	100
3	446468074	100	53	488808069	100
4	489343522	100	54	446988304	100
5	492912358	100	55	446791743	100
6	489499370	100	56	489504421	100
7	446137644	100	57	489346798	100
8	447025259	100	58	446568220	100
9	489497985	100	59	640582099	100
10	489073531	100	60	647819422	100
11	489495023	100	61	757558705	100
12	21957602	100	62	675757412	100
13	446013587	100	63	740811767	100
14	446271781	100	64	696235350	100
15	489818951	100	65	749299286	100
16	500999192	100	66	749300134	100
17	446461512	100	67	495569794	100
18	446540196	100	68	497549406	100
19	489818227	100	69	4972469	100
20	446087516	100	70	23305332	100
21	489181190	100	71	3882083	100
22	489498671	100	72	14335669	100
23	489204031	100	73	24106626	100
24	489175209	100	74	15638199	100
25	489343763	100	75	3043899	100
26	488139160	100	76	10716959	100
27	488942295	100	77	19879520	100
28	488142215	100	78	545013	100
29	446946313	100	79	15638493	100
30	446294093	100	80	28912358	100
31	489073924	100	81	22202881	100
32	489817928	100	82	22074017	100
33	446346290	100	83	19913144	100
34	446010456	100	84	17046717	100
35	488139900	100	85	29075682	100
36	489997344	100	86	15638505	100
37	446540198	100	87	15637937	100
38	491883924	100	88	28912298	100
39	446941606	100	89	28912382	100
40	489348577	100	90	12843514	100
41	489513185	100	91	15236674	100
42	445993072	100	92	28912268	100
43	489818381	100	93	12053733	100
44	11289046	100	94	18766230	100
45	488941687	100	95	5732209	100
46	447088107	100	96	300183	100
47	488144080	100	97	21628589	100
48	16416092	100	98	12855637	100
49	488941971	100	99	21954492	100
50	446625687	100	100	23598397	100

Table 10 Queries of length 500 for nr database

Id	Accession No.	Length	Id	Accession No.	Length
1	446136634	500	51	28950355	500
2	488940392	500	52	488150223	500
3	698982736	500	53	26334977	500
4	67469651	500	54	22477134	500
5	446111600	500	55	5734766	500
6	446377181	500	56	40316944	500
7	489497220	500	57	8163672	500
8	85110235	500	58	13508485	500
9	481023599	500	59	55622	500
10	446073879	500	60	124808622	500
11	526769928	500	61	7321134	500
12	526765571	500	62	17538500	500
13	694132388	500	63	446897307	500
14	696335236	500	64	6002620	500
15	740672164	500	65	4210322	500
16	686875550	500	66	23986207	500
17	686961337	500	67	29608200	500
18	495567824	500	68	10443291	500
19	495568938	500	69	14582421	500
20	17862970	500	70	254750635	500
21	148747522	500	71	17561336	500
22	9857716	500	72	1835208	500
23	18463969	500	73	29119276	500
24	28278907	500	74	21493015	500
25	21039040	500	75	1930065	500
26	29119286	500	76	14582441	500
27	26340652	500	77	27348214	500
28	499291684	500	78	32564943	500
29	18766383	500	79	15011369	500
30	21954316	500	80	29119693	500
31	14582429	500	81	20453805	500
32	7260620	500	82	1405817	500
33	446637732	500	83	23986221	500
34	22749021	500	84	32566151	500
35	29119677	500	85	9844871	500
36	5262157	500	86	32564038	500
37	21618274	500	87	575771805	500
38	19112286	500	88	14326568	500
39	75315260	500	89	3308978	500
40	496913	500	90	14582427	500
41	18491105	500	91	499365837	500
42	15991439	500	92	157869052	500
43	60193	500	93	18033652	500
44	29367581	500	94	29119691	500
45	28972954	500	95	4205009	500
46	12852969	500	96	950611	500
47	28875420	500	97	4205036	500
48	262263427	500	98	15011862	500
49	9105725	500	99	19912245	500
50	71274109	500	100	14537785	500

Table 11 Queries of length 1000 for nr database

Id	Accession No.	Length	Id	Accession No.	Length
1	688557372	1000	51	55139253	1000
2	27369652	1000	52	55168116	1000
3	14571749	1000	53	55726687	1000
4	22328898	1000	54	57530344	1000
5	21429096	1000	55	57869675	1000
6	4324748	1000	56	57869609	1000
7	240256190	1000	57	57899335	1000
8	124809435	1000	58	60360186	1000
9	124511784	1000	59	72387880	1000
10	15281471	1000	60	63098401	1000
11	5923681	1000	61	67553112	1000
12	4324739	1000	62	68062740	1000
13	22596379	1000	63	500101586	1000
14	436062	1000	64	494438949	1000
15	19075266	1000	65	500937396	1000
16	17533605	1000	66	500936235	1000
17	930252	1000	67	90812163	1000
18	22596309	1000	68	269316050	1000
19	17046663	1000	69	504836388	1000
20	22596515	1000	70	91200761	1000
21	29789203	1000	71	500600676	1000
22	24181509	1000	72	91089169	1000
23	19112190	1000	73	642933751	1000
24	21064719	1000	74	63014448	1000
25	499789111	1000	75	63014478	1000
26	568952968	1000	76	63014557	1000
27	32398993	1000	77	93354961	1000
28	39545749	1000	78	499846011	1000
29	33324437	1000	79	499853393	1000
30	922982901	1000	80	500591839	1000
31	37677875	1000	81	108862582	1000
32	37682421	1000	82	157133489	1000
33	37682471	1000	83	157133543	1000
34	37682589	1000	84	157113879	1000
35	37682791	1000	85	157136795	1000
36	37682510	1000	86	157120758	1000
37	389627648	1000	87	109090759	1000
38	38201798	1000	88	499887212	1000
39	1002292640	1000	89	500130697	1000
40	38892737	1000	90	145334361	1000
41	38892717	1000	91	111278795	1000
42	39777425	1000	92	112351529	1000
43	162287071	1000	93	113536096	1000
44	162138878	1000	94	1002307818	1000
45	148228726	1000	95	501243259	1000
46	1002269842	1000	96	501243094	1000
47	50312247	1000	97	115385747	1000
48	51860718	1000	98	115388543	1000
49	52626660	1000	99	115396338	1000
50	54650550	1000	100	114208152	1000

Table 12 Queries of mixed length for nr database

Id	Accession No.	Length	Id	Accession No.	Length
1	172072923	36	51	447111697	266
2	123964378	45	52	499767562	272
3	500811446	53	53	126325233	277
4	225785182	59	54	488313363	282
5	499467514	66	55	308810743	288
6	501552363	71	56	490745207	293
7	47059802	77	57	19684076	299
8	116067465	82	58	499879507	304
9	500839838	87	59	501120649	309
10	45593572	92	60	182636592	314
11	116275093	96	61	501046907	320
12	25986587	100	62	15242422	326
13	446409273	103	63	500480960	331
14	500931742	108	64	501328107	336
15	119447232	112	65	493847041	342
16	500027729	116	66	17562876	347
17	123185167	120	67	491518789	353
18	159113091	124	68	255920993	359
19	489451443	128	69	490659403	366
20	177841999	132	70	494504675	374
21	157937293	136	71	62131566	380
22	158703990	140	72	110004598	387
23	171687845	144	73	446784915	394
24	502110314	148	74	13736608	402
25	169154312	152	75	502299591	407
26	227204453	156	76	68300914	416
27	502646068	160	77	159491114	424
28	22036969	165	78	488693593	433
29	159031824	169	79	159111142	441
30	30267426	174	80	122956524	450
31	501188556	178	81	501214405	459
32	13924699	183	82	9663376	469
33	113529383	187	83	168203408	478
34	118025876	191	84	499852914	490
35	501240156	195	85	115492641	500
36	12657538	200	86	488242634	511
37	164597809	203	87	73853844	526
38	157929914	207	88	32479362	543
39	169819301	211	89	161377395	560
40	160917414	215	90	495078156	579
41	491993844	219	91	31127293	602
42	148566579	224	92	6522932	628
43	499922921	228	93	168049721	657
44	494373060	232	94	501130444	691
45	157082702	237	95	501642054	727
46	487814322	242	96	168024665	770
47	119708113	247	97	501445105	828
48	527104126	252	98	157836309	886
49	501987047	256	99	497563010	982
50	502141206	261	100	500116876	1127
